# Optimal frequency for magnetic resonant wireless power transfer in conducting medium

**DOI:** 10.1038/s41598-021-98153-y

**Published:** 2021-09-21

**Authors:** Thanh Son Pham, Thao Duy Nguyen, Bui Son Tung, Bui Xuan Khuyen, Thu Trang Hoang, Quang Minh Ngo, Le Thi Hong Hiep, Vu Dinh Lam

**Affiliations:** 1 Institute of Materials Science, Vietnam Academy of Science and Technology, 18 Hoang Quoc Viet, Cau Giay, Hanoi, Vietnam; 2 University of Science and Technology of Hanoi, Vietnam Academy of Science and Technology, 18 Hoang Quoc Viet, Cau Giay, Hanoi, Vietnam; 3 Graduate University of Science and Technology, Vietnam Academy of Science and Technology, 18 Hoang Quoc Viet, Cau Giay, Hanoi, Vietnam; 4 Fundamental of Fire Engineering Faculty, University of Fire Prevention and Fighting, 243 Khuat Duy Tien, Thanh Xuan, Hanoi, Vietnam

**Keywords:** Engineering, Physics

## Abstract

In this article, we investigated the efficiency of a magnetic resonant wireless power transfer (MR-WPT) in conducting medium and found out an optimal frequency for designing the system. In conducting environment, the eddy current loss is generated by the high-frequency alternating currents in the coils. It is manifested by increased radiation resistance of resonator coil leads to decrease the quality factor (*Q*-factor), which reduces the wireless power transfer (WPT) efficiency in conducting medium. The *Q*-factor of the resonator coil strongly depending on the conductivity, frequency, and thickness of conducting block. Two MR-WPT systems operating at 10.0 MHz and 20.0 MHz are implemented to study the effect of conducting medium on efficiency. The achieved results indicated that the 20.0 MHz system has higher efficiency at a conductivity smaller than 6.0 S/m. However, at the larger conductivity, the 10.0 MHz system is more efficient. The results provide a method to determine the optimal frequency of a WPT system operating in the conducting medium with various conductivities and thickness blocks. This method can be used to design MR-WPT systems in numerous situations, such as autonomous underwater vehicles and medical implants.

## Introduction

Recently, along with the proliferation of mobile electronic devices, wireless power transfer (WPT) is one of the new technologies that has a great development in both research and industry^1^. It has been applied to several areas in order to optimize convenience in daily human life^2,3^. The principle of the WPT, introduced by Nikola Tesla more than one century ago, is a process of transferring electrical energy from power sources to loads without conductor connection^4^. From the time of Tesla until now, the WPT has been considerably improved in the new transmission technique and scope of its applications^5^. Therefore, the WPT has great potential for several applications, such as mobile devices charging, electric vehicles, implantable devices, autonomous underwater vehicles (UAVs), space satellites, and so on^6–9^.

Compared to the radiative WPT, non-radiative WPT is based on the near-field interaction that is more suitable for consumer electronic devices, used in short and mid-range WPT (when the transmission distance is smaller than the wavelength)^10,11^. Among them, the inductive coupling-based WPT technique operates at kHz frequency bands widely used in short-range WPT applications^12^. However, a disadvantage of short-range WPT is that the transfer distance should be much less than the diameter of transmitter or receiver coils, limiting the applicability of recent WPTs^13^. Magnetic resonant wireless power transfer (MR-WPT), which operates at low MHz frequency bands, demonstrates a longer transmission length compared to the inductive coupling method^14^. Typically, MR-WPT has a 4-coil configuration, including a source loop, a load loop, and two high-quality factor resonators. Thanks to the magnetic energy concentrated in high-quality factor resonators, the energy can be transferred efficiently from the transmitter to the receiver end^15–18^.

Most research and applications of WPT mainly focus on transferring energy in the air or non-conducting medium. The potential of WPT is not limited by these environments, therefore it can also be applied in specific environments such as underground, underwater, and transference through tissues. WPT simultaneously transmits power and information underground with better efficiency than conventional antenna systems have demonstrated by Ma et al*.*^19^. Another wireless charging system that transfers power and signal for autonomous underwater vehicles (UAVs) was developed in Ref.^20^. Moreover, applying the WPT technique to biomedical devices implanted inside the human body, where the power wirelessly transfers through the tissue, was investigated^21^. Unlike in the air, these above media can be electrically conductive. Therefore, WPT suffers a significant loss from eddy current^22^. The previous study investigated the effect of conductive medium on the coupling between transmitter and receiver in WPT systems, analyzed eddy current with misalignment, and eddy current loss dependency on frequency^23–26^. However, these studies mainly focus on inductive coupling WPT systems operating at kHz range frequency for short transfer distances. The demand of the increasing transferring range requires a comprehensive analysis for MR-WPT in conducting medium. The relationship between radiation resistance generated by the eddy current and *Q*-factor of resonator that affects the transfer efficiency needs to be considered.

In this paper, we investigate the MR-WPT system, which transfers power through a conducting medium at low MHz frequency range. The external radiation resistance of resonator due to the eddy current is considered, which causes the loss and reduces the *Q*-factor of resonator. The simulation indicates that the eddy current is generated in the conducting block place in between transmitter (Tx) and receiver (Rx) resonators. Depending on the conductivity and thickness of conducting block, there is an optimal frequency where the resonator reaches the maximum *Q*-factor. Thanks to that, the WPT system gives the highest transfer efficiency. The experiment is performed to confirm the effect of conductivity, frequency, and thickness of conducting block on the efficiency. The results of this study provide an effective method to determine the optimal frequency of MR-WPT systems in conducting medium with different conductivities and thickness blocks.

## Results

### Radiation resistance in conducting medium

Considering the WPT applications in the air, the Ohmic loss caused by the resistance of the coil is dominant, while the radiation causes a loss that can be neglected at the low-frequency operation band of MR-WPT^27^. However, in the conducting medium, the magnetic field variation at high frequency in the resonator coils generates an eddy current in the surrounding medium, which influences the transfer efficiency. The loss from this current must be considered. Therefore, the transfer efficiency of WPT in the conductive medium can be expressed as^23^

1 $$\eta_{cond} = \frac{{P_{out} }}{{P_{out} + I_{T}^{2} R_{T} + I_{R}^{2} R_{R} + P_{eddy} }}$$

where (*I*_T_, *I*_R_) and (*R*_T_, *R*_R_) are the currents and resistances in the air of the transmitter and receiver, respectively, $P_{out} = \omega_{0} MI_{T} I_{R}$ is the output power, *M* is the mutual inductance between the transmitter and the receiver^22^. *P*_eddy_ is the loss caused by the eddy current, which can be determined by the radiation resistance of coil in the conducting medium^28^. Therefore, the transfer efficiency of WPT in the conductive medium can be calculated as

2 $$\eta_{cond} = \frac{{P_{out} }}{{P_{out} + I_{T}^{2} R_{T} + I_{R}^{2} R_{R} + I_{T}^{2} R_{T - rad} + I_{R}^{2} R_{R - rad} }}$$

where *R*_T-rad_ and *R*_R-rad_ are radiation resistance of transmitter and receiver, respectively.

Assume that system is symmetric, the transmitter and the receiver are identical, and the transfer efficiency is maximized when *I*_T_ = *I*_R_^22^. Therefore, the maximum transfer efficiency of WPT can be rewritten as

3 $$\eta_{cond}^{\max } = \frac{1}{{1 + \frac{2}{{kQ_{cond} }}}}$$

where *k* = *M*/*L* is the coupling coefficient, *L* is the inductance of resonator, *Q*_cond_ is the quality factor of the transmitter and receiver in the conducting medium, which can be defined by

4 $$Q_{cond} = \frac{\omega L}{{R + R_{rad} }}$$

As a result of Eq. (3), the maximum transfer efficiency depends on *kQ*_cond_ product but not on the power level. Power level is proportional to the power loss by eddy current. However, the ratio *P*_eddy_/*P*_out_ is independent of the power level and affected by the resonant frequency and system dimension. At the given distance, the coupling coefficient *k* is fixed, a higher quality factor *Q*_cond_ results in higher maximum transfer efficiency.

Next, we consider the resistance of a coil, which parameter strongly affects the *Q*-factor of a resonator. The total resistance of a coil in the air or conductive medium is given by

5 $$R = R_{DC} + R_{AC} + R_{rad}$$

where *R*_DC_ and *R*_AC_ are the *DC* and *AC* resistance of the coil, *R*_rad_ is the radiation resistance. Although *R*_DC_ depends on the coil size and *R*_AC_ on skin depth, they are similar, but the radiation resistance has a large difference at the high frequency in the air and conducting medium.

The external radiation resistance for a loop in a conducting medium can be calculated by^28^

6 $$R_{rad}^{cond} = \omega \mu r\left[ {\frac{4}{3}\left( {\beta r} \right)^{2} - \frac{\pi }{3}\left( {\beta r} \right)^{3} + \frac{2\pi }{{15}}\left( {\beta r} \right)^{5} - ...} \right]$$

where *ω* is the angular frequency, *µ* is the permeability of medium, *r* is the radius of loop in meters and *β* = (*µωσ*/2)^1/2^ with *σ* is the conductivity of the medium.

The radiation resistance of a single loop in the air given by^28^

7 $$R_{rad}^{air} = \frac{\pi }{6}\frac{{\omega^{4} \mu r^{4} }}{{c^{3} }}$$

where *c* is the velocity of light in free space. In comparison, the radiation resistance of a loop in the air is much smaller than that in conducting medium. Therefore, the radiation loss of a WPT system in conducting medium is significant and needs to be considered.

### Eddy current

**Figure 1:**
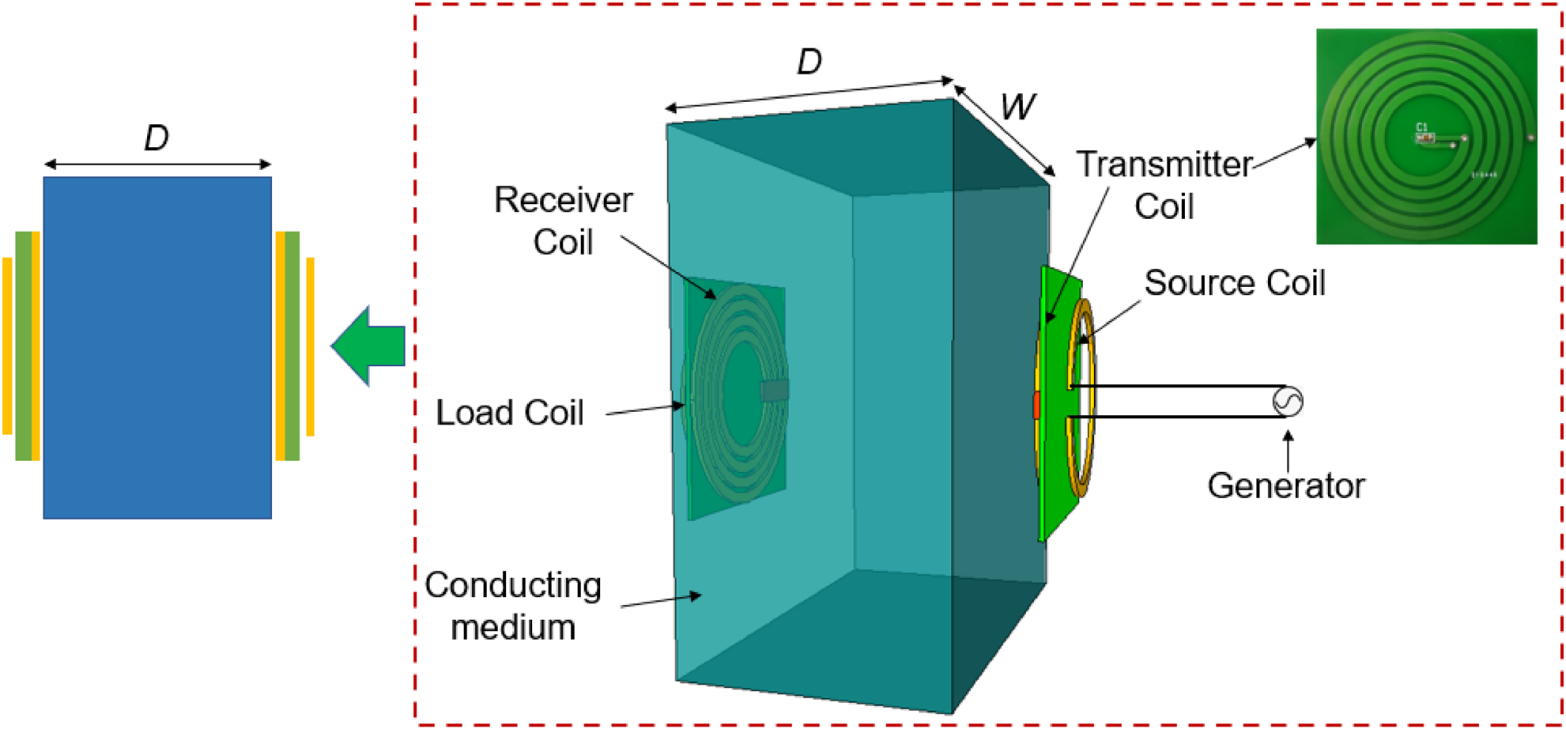
Schematic of MR-WPT system with a conducting block in the between Tx and Rx.

As aforementioned, the efficiency of WPT system is influenced by the medium’s conductivity due to the radiation resistance generated by the eddy current. To investigate the effect of conducting medium on the efficiency, we perform an MR-WPT system, transferring energy through a conducting block with the thickness of *D* and width of *W*, as illustrated in Fig. 1.

**Figure 2:**
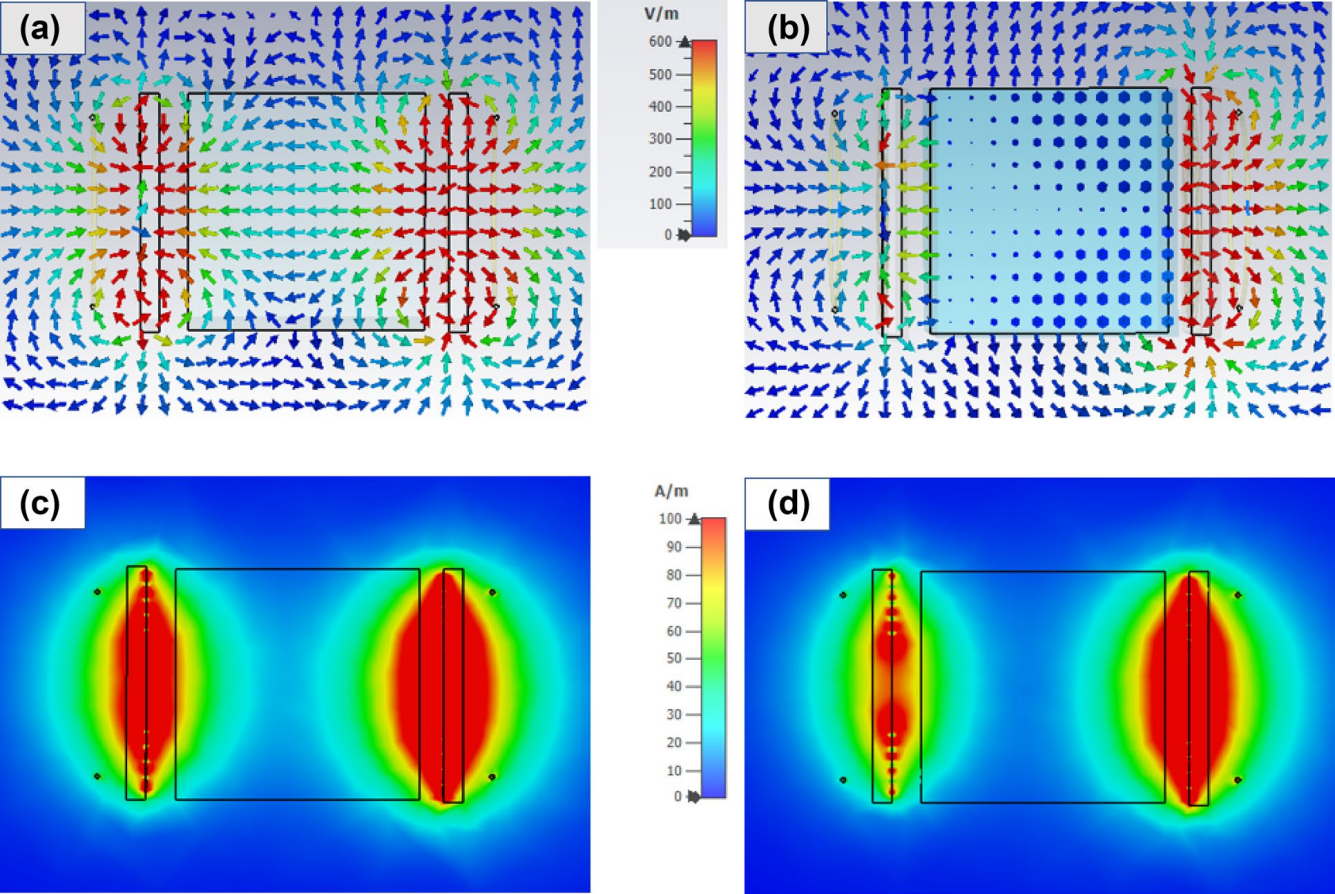
Field intensity distribution of **(a)** E-field in free space, **(b)** E-field with a conducting block, **(c)** H-field in free space, **(d)** H-field with a conducting block.

Figure 2 shows the simulation results obtained by the Computer Simulation Technology Microwave Studio (CST-MWS) for field distribution in the MR-WPT system with and without conducting block at the frequency of 20.0 MHz. Figure 2a,b show the E-field, and Fig. 2c,d show the H-field around the WPT system in free space and presenting a conducting block with *D* = 5.0 cm and *W* = 5.0 cm, respectively. The conductivity of the medium is 4.0 S/m, it is equivalent to the conductivity of seawater. In the free space case, the E-field is concentrated at position near the resonator coil and is degraded at a location far from the coil. The E-field generated by the Tx coil is linearly polarized. The arrows demonstrate that the strength and direction of the fields are seamless, as shown in Fig. 2a. In Fig. 2b, out of the conducting box, the E-field is similar to the free space, but the strength of the E-field is degraded in the conducting box, and the E-field direction changed compared to the free space case. At the location near the Rx resonator, the E-field strength is reduced when the conducting block appears. In Fig. 2c,d, the H-field strength near the Rx in free space is stronger than that when presenting conducting block, which is similar to the E-field case. However, the H-field is continuous across the boundary of conducting block. Both E-field and H-field strength at the location near the Rx resonator are degraded when presenting the conducting block, which leads to a reduction of WPT efficiency.

**Figure 3:**
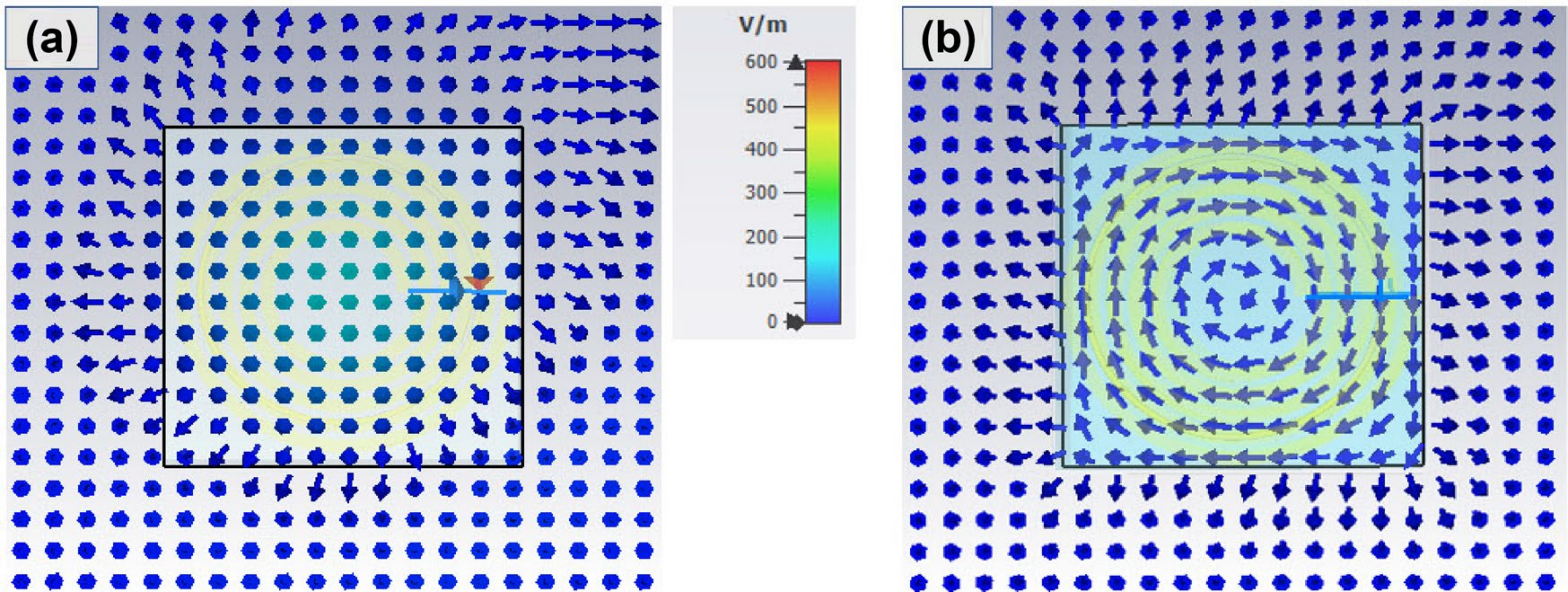
Electric field intensity distribution at the middle plane of **(a)** free space and **(b)** with a conducting block.

Figure 3a,b show the E-field intensity distribution at the middle plane of MR-WPT system. In the free space, representative arrows for E-field have one direction from Tx to Rx. Some arrows have slightly different directions because of the geometry of Tx and Rx generating a non-uniform field. Interestingly, when the conducting block is presented, eddy currents are excited inside the block and produce a reaction E-field. All the arrows inside the box change their direction and are in the form of a vortex. This observed eddy current is the origin of the extra loss when transferring power through a conducting medium.

### Conducting medium effect to resonator’s quality factor

The resonator coil in WPT system can be modeled as an equivalent *RLC* circuit, as shown in Ref.^18^. The system can be represented as a one-port network. Therefore, the properties of the resonator can be expressed in terms of network parameters, such as *S, Z*, *Y*, and *ABCD*^29^. From the definition of Z-parameters, the input impedance of the resonator coil is given by

8 $$Z_{in} = R + j\omega L + \frac{1}{j\omega C}$$

As the resonator coils in an inductive link are operated below self-resonant frequency, *RL* model is sufficiently accurate to replace *RLC* model^30^. The resistance and the inductance of resonator could be extracted from the real part of *Z*_in_ and its reactance. The *Q*-factor of resonator coil can be determined from the resistance and inductance by

9 $$Q = \frac{\omega L}{R}$$

**Figure 4:**
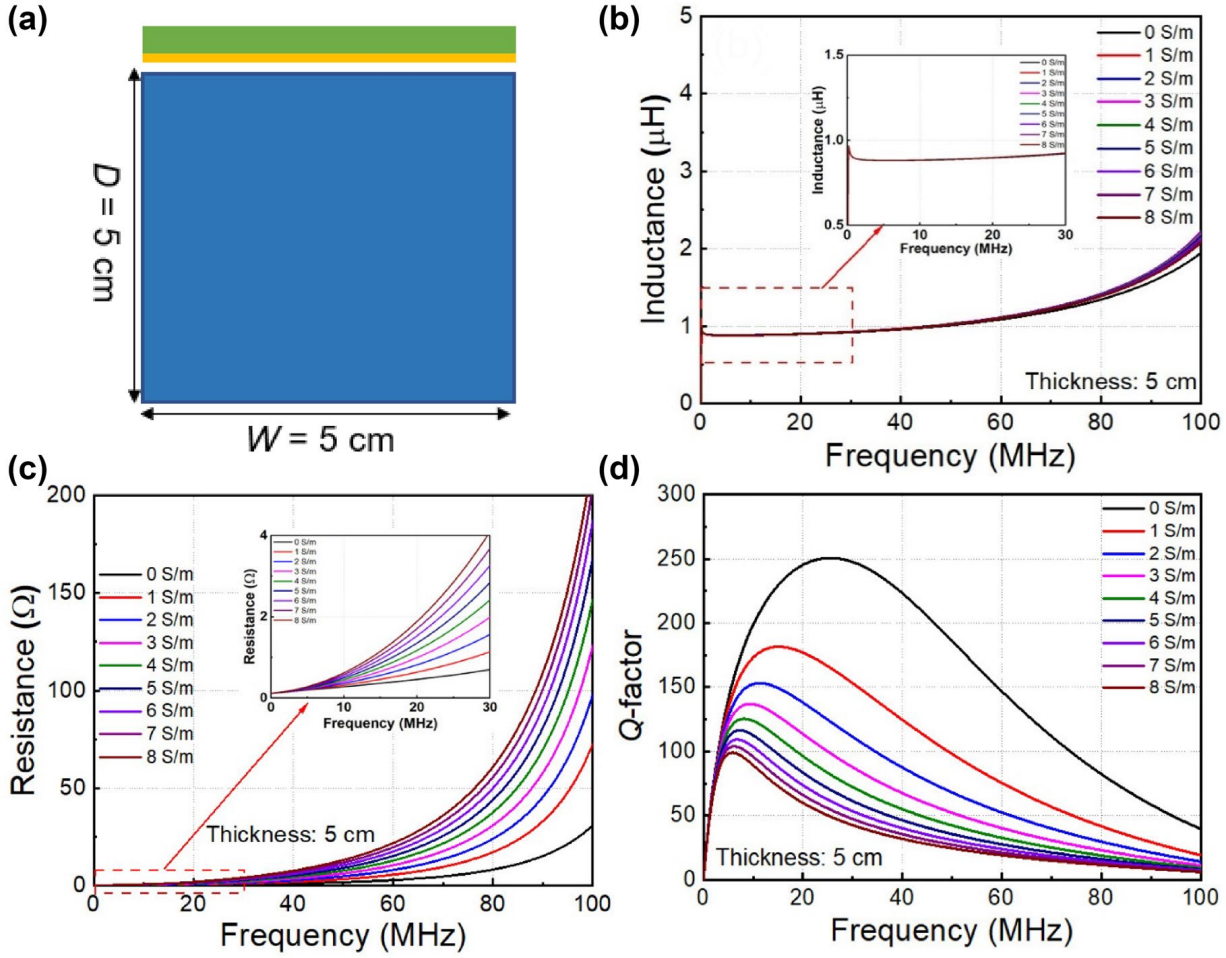
Resonator coil parameters as a function of frequency at various conductivities, **(a)** schematic view of the simulation setup, **(b)** inductance, **(c)** resistance, **(d)** quality factor.

Figure 4a depicts the schematic view of the simulation setup to investigate the resonator coil, where it is placed close to the conducting block with the thickness *D* = 5.0 cm, width *W* = 5.0 cm. The detailed parameters of resonator coil are listed in Table 2 (5 cm diameter) in “Methods” section. When the resonator coil is immersed in conducting medium, its impedance is changed because of the eddy current. Figures 4b–d show the parameters of resonator coil following the change of conductivity when the conducting block thickness is *D* = 5.0 cm. The inductance of resonator coil is changed slightly at the frequency range from zero to 30.0 MHz (see in the inset), as shown in Fig. 4b. We focus on frequency range from 0 to 30.0 MHz because most MR-WPT systems are designed in that band. Figure 4c shows the resistance of resonator coil at different conductivities. In the non-conducting medium or air, the total resistance is increased as the frequency due to the skin depth effect (increasing *R*_AC_), as shown by the black curve. When the medium is conductive and at the same frequency, the total resistance of resonator coil is strongly increased with conductivity. The increase of total resistance can be explained by the external radiation resistance in conducting medium of resonator coil. It can also be described as the loss by eddy current in the conducting medium. Therefore, the loss increases when the medium’s conductivity is high, leading to increased resistance when conductivity changes from 1.0 to 8.0 S/m. Accordingly, as the frequency also affects the eddy current loss, the resistance tends to be larger at higher frequencies. From the results of inductance and resistance, we can calculate the *Q*-factor of resonator coil. The *Q*-factor is a critical parameter to determine the efficiency of WPT system. Figure 4d shows the *Q*-factor of resonator coil as a function of frequency at different medium conductivities. The increase in resistance (while inductance is slightly changed) decreases the *Q*-factor of the resonator coil with increasing conductivity. Interestingly, the *Q*-factor reduction rate at different frequencies is numerous, leading to the frequency obtained maximum *Q*-factor shifts to a lower range when conductivity is increased.

**Figure 5:**
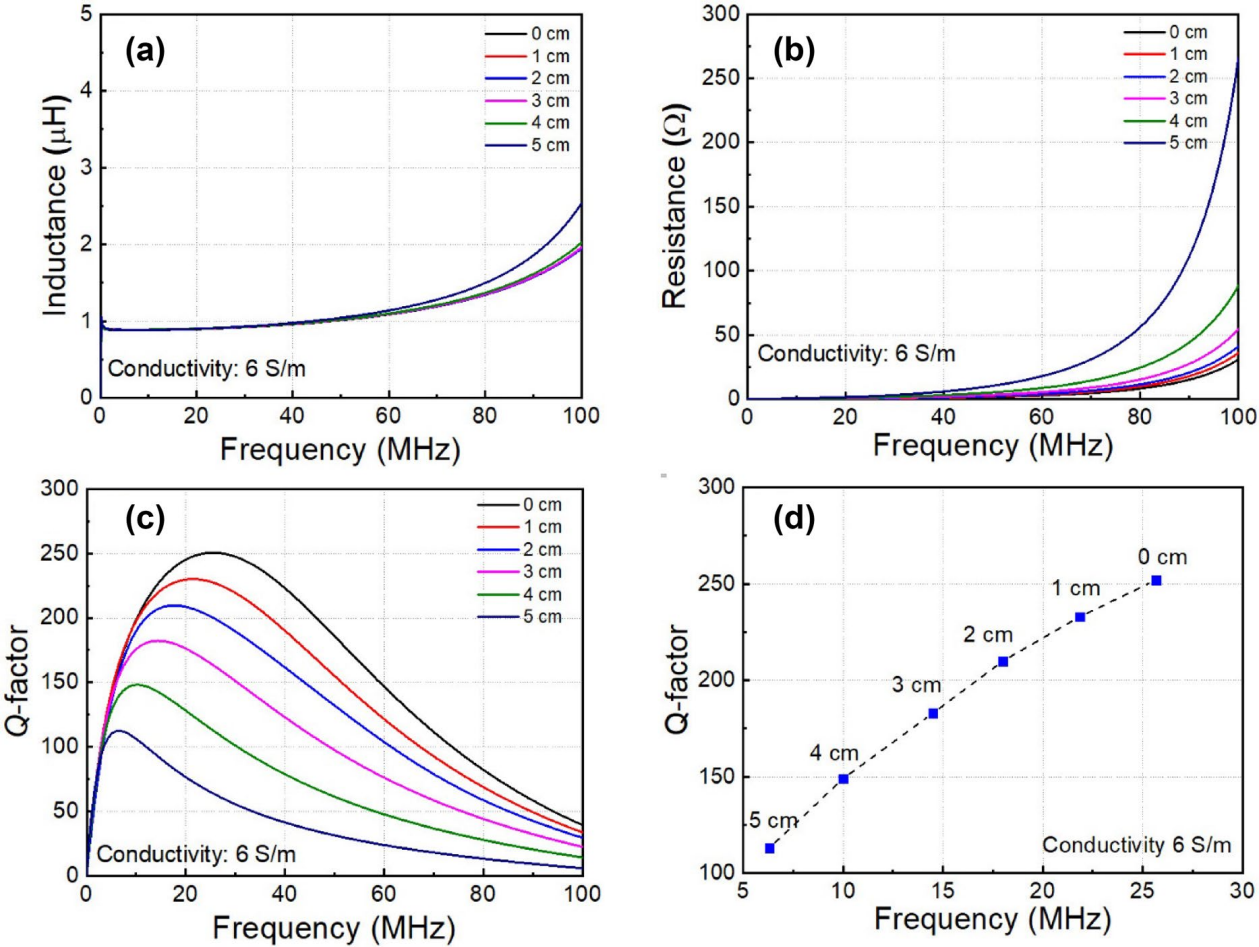
Resonator coil parameters as a function of frequency at different conducting block thickness, **(a)** inductance, **(b)** resistance, **(c)** quality factor, **(d)** peak quality factor.

Next, we consider the key parameters of resonator with various conducting block thicknesses. Figure 5 shows the parameters *L*, *R*, *Q* of resonator when the conducting block thickness changes from 0 to 5.0 cm, and the conductivity is kept at 6.0 S/m. Indeed, an increase in thickness means a larger volume for eddy currents. Hence the total resistance of the resonator coil is increased (Fig. 5b) while the inductance is slightly changed (Fig. 5a), resulting in a decrease in the *Q*-factor (Fig. 5c). With increases in conducting block thickness, the frequency to achieve the maximum *Q*-factor of resonator is also reduced from 25.6 to 6.5 MHz, as shown in Fig. 5d. This result implies that a WPT system through a conducting medium has a specific frequency for achieving maximum efficiency. Therefore, the larger thickness of conducting block requires a smaller optimal frequency.

## Experiment results

**Figure 6:**
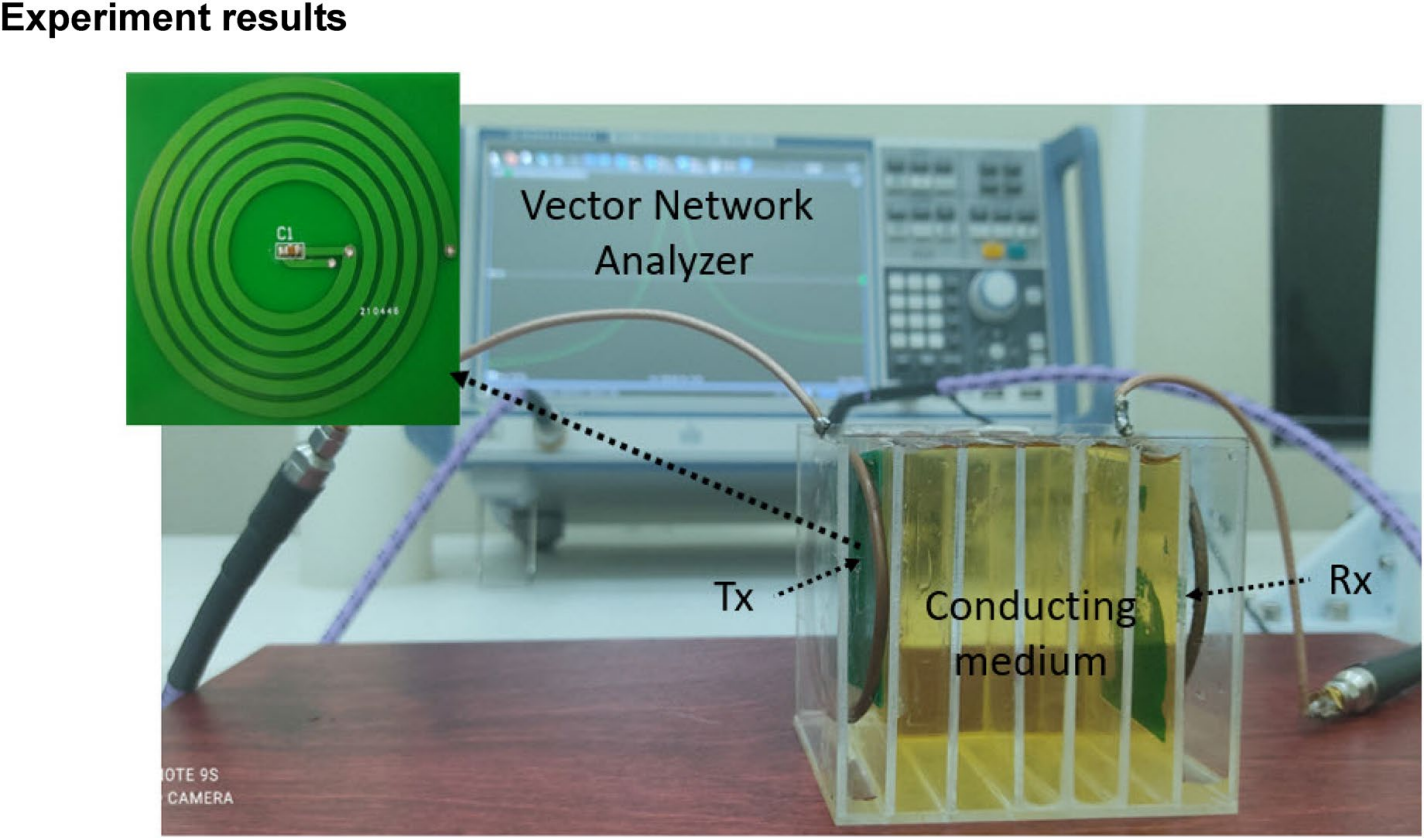
Experimental setup for MR-WPT in conducting medium.

To confirm analysis in previous sections, we experimentally characterized the transfer efficiency of proposed MR-WPT system with a conducting block of 5.0 cm thickness, as shown in Fig. 6. The configuration of MR-WPT system is similar to the simulation part in Fig. 1. The transmitter and receiver parts are fabricated by the PCB technique, thus limiting fabrication errors and easy replication. We investigate the MR-WPT system at two operating frequencies 10.0 MHz and 20.0 MHz. The operating frequency of MR-WPT system is defined by the resonant frequency of resonator. The dimensions of MR-WPT system in both configurations were kept to be the same. To change the operating frequency, we only adjusted the value of external capacitors embedded in the resonator coils (261 pF for 10.0 MHz and 50 pF for 20.0 MHz). The detailed parameters of MR-WPT system are listed in Table 2 in the Methods section.

**Figure 7:**
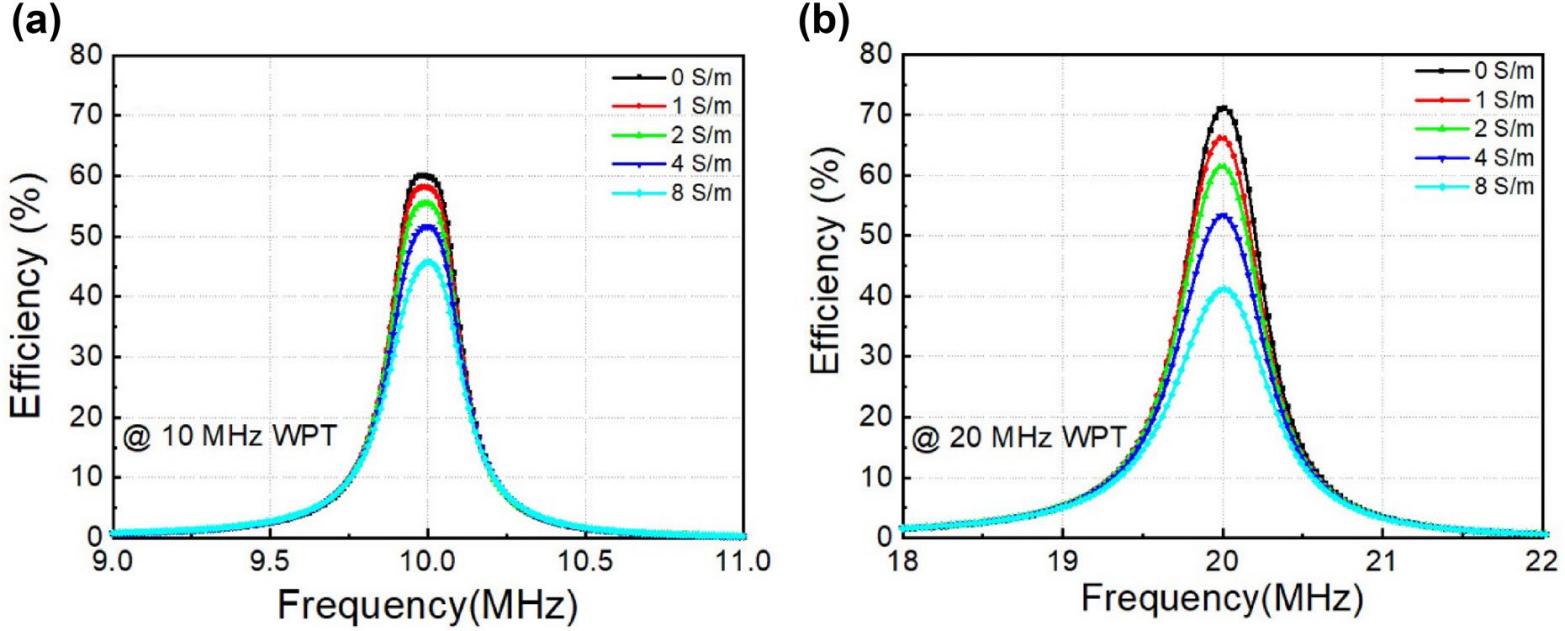
Measured transfer efficiency as a function of frequency of MR-WPT system with various conductivity, **(a)** operating frequency at 10.0 MHz, **(b)** operating frequency at 20.0 MHz.

Figure 7a shows the efficiencies as a function of frequency of proposed MR-WPT systems operated at 10.0 MHz with various conductivities. In pure water, this is a normal MR-WPT system, which provides an efficiency of 60%. When the conductivity rises to 1.0, 2.0, 4.0 and 8.0 S/m the efficiency decreases to 58%, 55%, 51% and 45%, respectively. The increase in conductivity leads to the decline of transfer efficiency because of eddy currents in the conducting block, as indicated in the above analysis. Figure 7b shows the results of the similar experimental setup at the operating frequency 20.0 MHz. In pure water, the *Q*-factor of the resonator coil at 20.0 MHz is larger than that at 10.0 MHz. Therefore, in this case, the obtained results confirm the efficiency of 70% (compared to the efficiency of 60% in the previous system). This implies that in pure water or free space using 20.0 MHz WPT is better than 10.0 MHz WPT. On the other hand, the trend is opposite in conducting medium, where the efficiency declines faster at higher frequency in comparison with that at lower frequency ranges. This is a result of the increase in the external radiation of the resonator in the conducting medium, as represented through the eddy current at the higher frequency. The efficiency reduces to 40% at the conductivity of 8.0 S/m in 20.0 MHz MR-WPT system, from the observed 45% at 10.0 MHz. It can be noted that the efficiency of the proposed MR-WPT system is smaller than previous work^22^ at the similar conducting block thickness. However, the coil dimension is miniaturized to be only 5.0 cm, which is smaller than their value of 17.6 cm.

**Figure 8:**
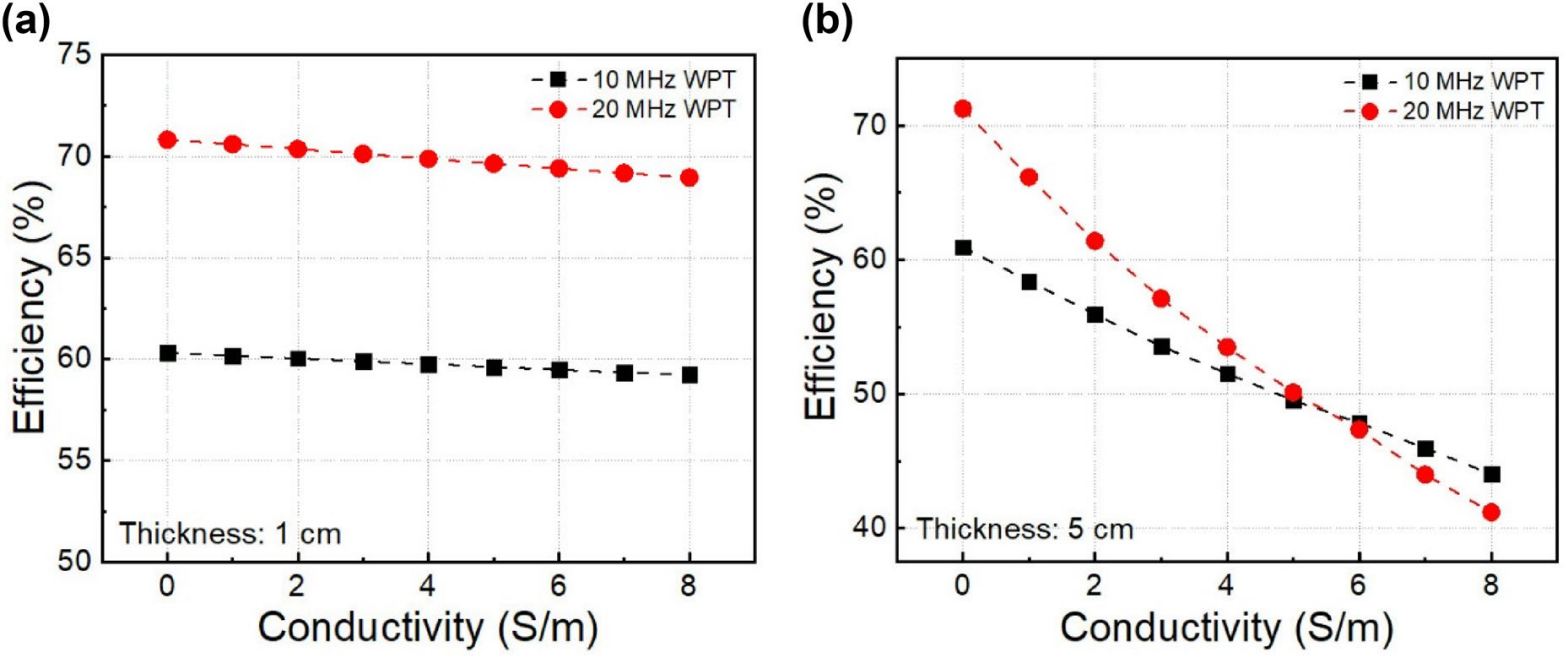
Measured transfer efficiency of two MR-WPT systems as a function of conductivity, **(a)** conducting block thickness of 1.0 cm, **(b)** conducting block thickness of 5.0 cm.

Besides the dependence conductivity, the transfer efficiency is also affected by the thickness of conducting block. Figure 8a shows the efficiency when the thickness of conducting block is kept at 1.0 cm and the conductivity is changed from 0 to 8.0 S/m. In these configurations, the distance between transmitter and receiver is 5.0 cm, and the conducting block is placed between the transmitter and receiver. At the same conductivity, 20.0 MHz WPT system achieves higher efficiency than 10.0 MHz WPT system. As expected, the efficiencies of both systems are decreased gradually by increasing the conductivity. The efficiency of 20.0 MHz MR-WPT system drops faster, but there is no significant difference. The thickness of the conducting block is set at 5.0 cm in Fig. 8b. Because of more dissipated energy in the conducting medium, the efficiency of MR-WPT is significantly decreased with both 10.0 MHz and 20.0 MHz. However, at 20.0 MHz, the efficiency is decreased much faster compared with 10.0 MHz. At small conductivities, the efficiency of 20.0 MHz WPT system is higher, but the efficiency of 10.0 MHz WPT system tends to be better when the conductivity is more than 6.0 S/m. This result denotes the trade-offs when designing MR-WPT systems in the conducting media. The frequency for achieving maximum *Q*-factor of the resonator and thus obtaining the highest transfer efficiency is lower when increasing the conductivity and thickness of conducting block.

**Figure 9:**
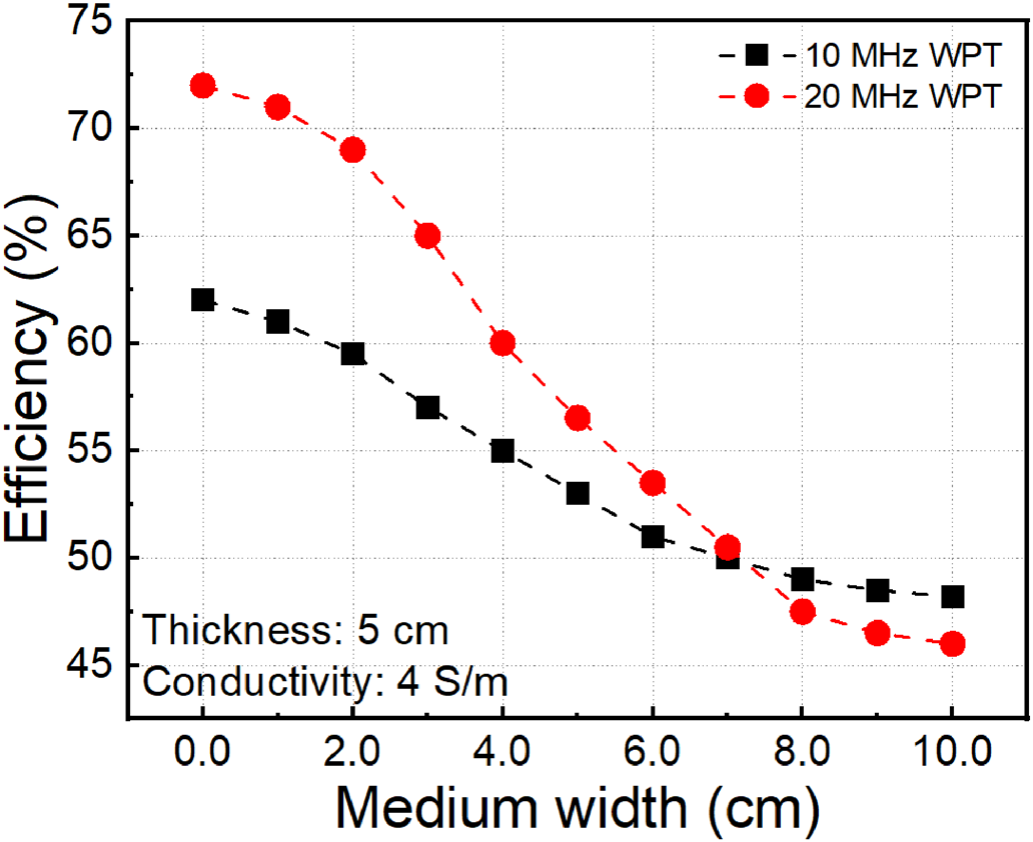
Measured transfer efficiency of two MR-WPT systems as a function of medium size.

The power loss increase with the size of conducting medium, which reduces the transfer efficiency. Figure 9 shows the transfer efficiency of MR-WPT systems depending on the width of conducting medium at the conductivity is 4 S/m and the thickness of conducting block of D = 5.0 cm. The results indicate that the efficiencies of both systems are decreased gradually by increasing the medium size. However, the 20.0 MHz MR-WPT is more affected by the size of the conducting medium and leads to a more significant efficiency drop. When the size of conducting medium W > 7.0 cm, the attenuation reaches to saturation regime, and the efficiency of 10.0 MHz MR-WPT system is better than 20.0 MHz MR-WPT system.

**Figure 10:**
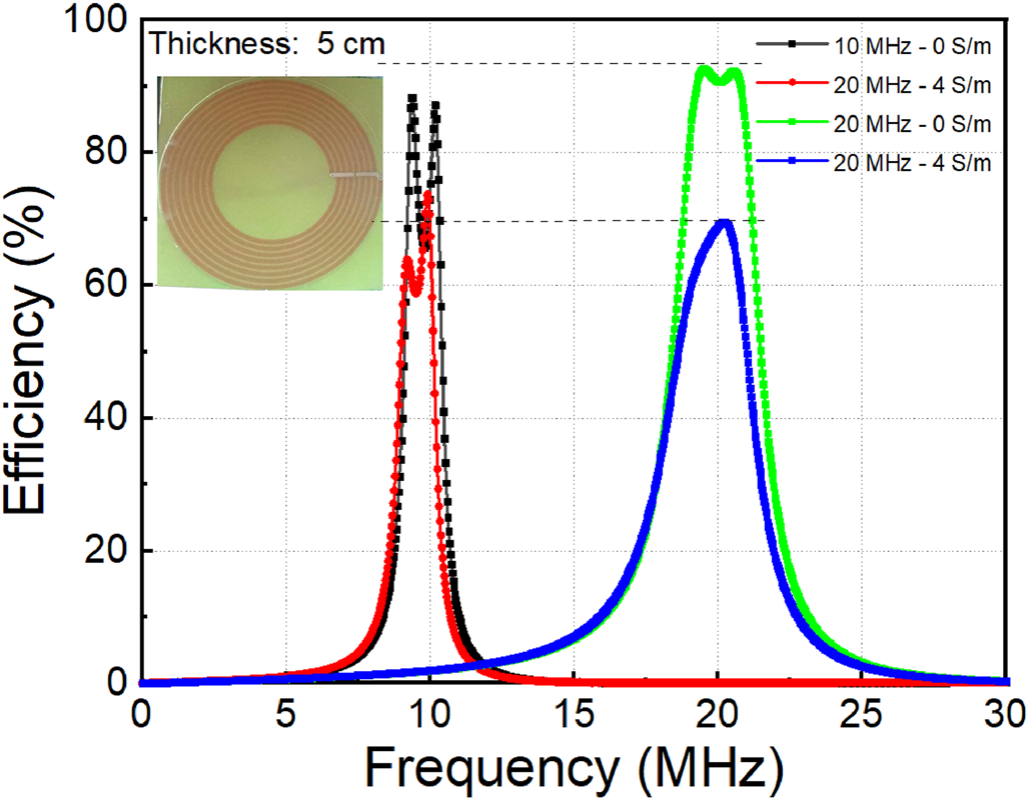
Measured transfer efficiency of two MR-WPT systems with resonator diameter of 10.0 cm.

To investigate the effect of resonator size on the transfer efficiency, we consider an MR-WPT system with a resonator diameter of 10.0 cm. The detailed parameters of this MR-WPT system are list in Table 2 in the Methods section. The size of the coil is larger, therefore to adjust the resonant frequency of resonator the different capacitor values are chosen to create the 10.0 MHz and 20.0 MHz MR-WPT systems (140 pF for 10.0 MHz MR-WPT and 30 pF for 20.0 MHz MR-WPT). Figure 10 shows the transfer efficiency of MR-WPT systems at the conductivities of 0 (free space) and 4 S/m. In this configuration, the width (*W* = 10.0 cm) of conducting block is equal to the diameter of resonator coil. Because of the larger size of resonator (10.0 cm of diameter) compared to the transfer distance (5.0 cm), the MR-WPT system operates within the over-coupled region^5^. The transfer efficiency spectrum is split into two peaks on either side of the original resonant frequency. The peak efficiency of 20.0 MHz system at the free space is 92% compared to 88% of 10.0 MHz system. In this configuration, the efficiency is higher than the previous (the MR-WPT with 5.0 cm diameter of resonator) due to the diameter is double (10.0 cm compared to 5.0 cm) while the transfer distance is kept the same of 5.0 cm. When the conducting block is placed, the transfer efficiencies of both systems are decreased similarly to the previous cases. However, the efficiency of 20.0 MHz system is lower than 10.0 MHz system (70% compared with 75%), whereas it is the opposite in the case of 5.0 cm of resonator diameter (56% compared with 53%). The results show that the degradation of transfer efficiency is faster at larger resonator sizes. That can be explained by larger radiation resistance when the diameter of coil increases.

## Discussion

In this work, we demonstrate an MR-WPT system in the conducting medium where the external radiation resistance of the resonator coil increases in frequency and conductivity. Therefore, the *Q*-factor of the resonator coil decreases in the conducting medium, whereas the optimized frequency to achieve this maximum *Q*-factor can be controlled under different conditions. In the conducting medium, the effect of eddy current on power loss can be described by radiation resistance and *Q*-factor of resonator. Depending on the conductivity and thickness of the conducting block, the optimal operating frequency of MR-WPT system for obtaining the maximum transfer efficiency is particular. At the small conductivity, a higher operating frequency is better. However, with larger conductivity, the lower frequency is more suitable. The dependence of transfer efficiency on the thickness, width of the conducting block, and resonator size was also investigated. This work provides an understanding of frequency optimizing of the MR-WPT system operating in the conducting medium. The result can be applied to design MR-WPT for the implant devices, under seawater vehicles, underground devices. Compared to the inductive coupling WPT, which has widely used in such applications, MR-WPT achieves a significant efficiency improvement at the same transfer distance per coil diameter ratio. Therefore, which can increase the transfer range and reduce the system dimension.

Transferring energy through the conducting medium is a challenging task due to its attenuation characteristics. Several approaches have been implemented to solve this problem. The inductive coupling WPT^22,26,32^ were used in the kHz frequency range and achieved high transfer efficiency, but these transfer distances are small compared to the coil diameters. WPT in GHz frequency range^21^ was also used to transfer energy through deep tissue with small efficiency. Some previous works study resonant coupling WPT in both kHz^20^ and low MHz^31^ frequency range, but they did not mention the dependence of frequency and conductivity to transfer efficiency. For each specific conductivity, the operating frequency of the WPT system must be different to achieve the highest efficiency. The detailed performance comparisons of WPT systems in the conducting medium are shown in Table 1.

**Table 1:** Performance comparisons of WPT system in conducting medium.

Topylogy	Frequency	Conductivty	Transfer distance (cm)/coil diameter (cm)	Efficiency (%)	References
Resonant coupling	0.65 kHz	5 S/m	170/340 (0.5)	59	^20^
GHz WPT	1–4 GHz	Tissue	2/1–6 (2–0.33)	0.4–0.004	^21^
Inductive coupling	70–520 kHz	4 S/m	6.6/17.6 (0.38)	81–90	^22^
Inductive coupling	0.1–1 MHz	4.5 S/m	0.5/21 (0.023)	95–35	^26^
Resonant coupling	2.12 MHz	4 S/m	4/6 (0.67)	53	^31^
Inductive coupling	80 kHz	4 S/m	1.6/6 (0.27)	75	^32^
Resonant coupling	10 MHz, 20 MHz	1–8 S/m	5/5 (1)	66–42	This paper

## Methods

### Transmitter and receiver design and fabrication

The transmitter part of MR-WPT consists of a source loop that has a diameter of 4.0 cm and a five-turn spiral resonator. The resonator in this work has a circular shape with a diameter of 5.0 cm, a strip width of 2.5 mm, and an inter-strip space of 1 mm, with a copper thickness of 0.105 mm. This structure is fabricated on the FR-4 substrate with a thickness of 1.0 mm has a dielectric constant of 4.4. The resonant frequency of resonator can be controlled by an external capacitor embedded in the gap of two spiral ends. The receiver part has the same structure and composition as the transmitter part.

To investigate the effect of resonator size on the performance, an MR-WPT system with larger resonators is designed with the same procedure as above. The parameters of MR-WPT systems are listed in Table 2.

**Table 2:** Design parameters of WPT systems.

System parameter	5.0 cm diameter	10.0 cm diameter
Source-load loop diameter	4.0 cm	8.0 cm
**Tx–Rx resonator**		
Number of turn	5	8
Diameter	5.0 cm	10.0 cm
Strip width	2.5 mm	3.0 mm
Inter-strip space	1.0 mm	1.0 mm
FR-4 thickness	1.0 mm	1.0 mm
Capacitor for 10.0 MHz WPT	261 pF	140 pF
Capacitor for 20.0 MHz WPT	50 pF	30 pF
Transmission distance	5.0 cm	5.0 cm

### Efficiency measurement

After the standard two-port calibration process, the scattering parameters are measured using the Vector Network Analyzer (VNA—Rohde & Schwarz ZNB20). At the resonant frequency, the distance between the source loop and transmitter (load and receiver) was adjusted using the variable coupling method^33^ until both *S*_11_ and *S*_22_ were less than −10 dB. This ensures that the effect of the impedance mismatch is removed from the measurement setup. Therefore, the transfer efficiency of MR-WPT system can be calculated using the measured |*S*_21_|^2^. The error of experimental results is less than 1% for all measurements.

### EM simulations

All the simulation results are obtained using a commercially available, finite element method solver-based EM simulator, Computer Simulation Technology Microwave Studio (CST-MWS) software. The magnetic field distribution is achieved at the operating frequency of MR-WPT system. Inductance, resistance extracted using post-processing tool in CST software^34^.
